# Co-Authorship and Keyword Networks in Five Decades of Molecular Simulation: A Network Analysis of a Scientific Publication Portfolio

**DOI:** 10.1021/acs.jpcb.6c05147

**Published:** 2026-10-26

**Authors:** Livia B. Pártay, Ottilia Fülöp, Béla Barabás

**Affiliations:** † Department of Chemistry, 2707 University of Warwick, Coventry CV4 7AL, U.K.; ‡ Department of Analysis and Operations Research, Institute of Mathematics, 61810 Budapest University of Technology and Economics, Müegyetem rkp. 3, Budapest 1111, Hungary; § HUN-REN Alfréd Rényi Institute of Mathematics, Reáltanoda u. 13-15, Budapest 1053, Hungary; ∥ Department of Stochastics, Institute of Mathematics, Budapest University of Technology and Economics, Müegyetem rkp. 3, Budapest 1111, Hungary

## Abstract

In the present paper, we illustrate and analyze the coauthorship network of publications authored by Michael L. Klein using standard social network analysis techniques. Beyond presenting and analyzing the author-keyword network assigned to his publications, we also introduce abstract-similarity networks to enable a deeper and broader exploration of the relationships among Michael L. Klein’s publications. The resulting network structures and communities are also interpreted from a chemical perspective, highlighting recurring connections between simulation methodology, force-field development, and major application areas such as aqueous systems, lipid membranes, membrane proteins, and soft-matter self-assembly. More broadly, the study illustrates how network-based approaches can be used to map the organization of a large molecular simulation research program.

## Graphical abstract

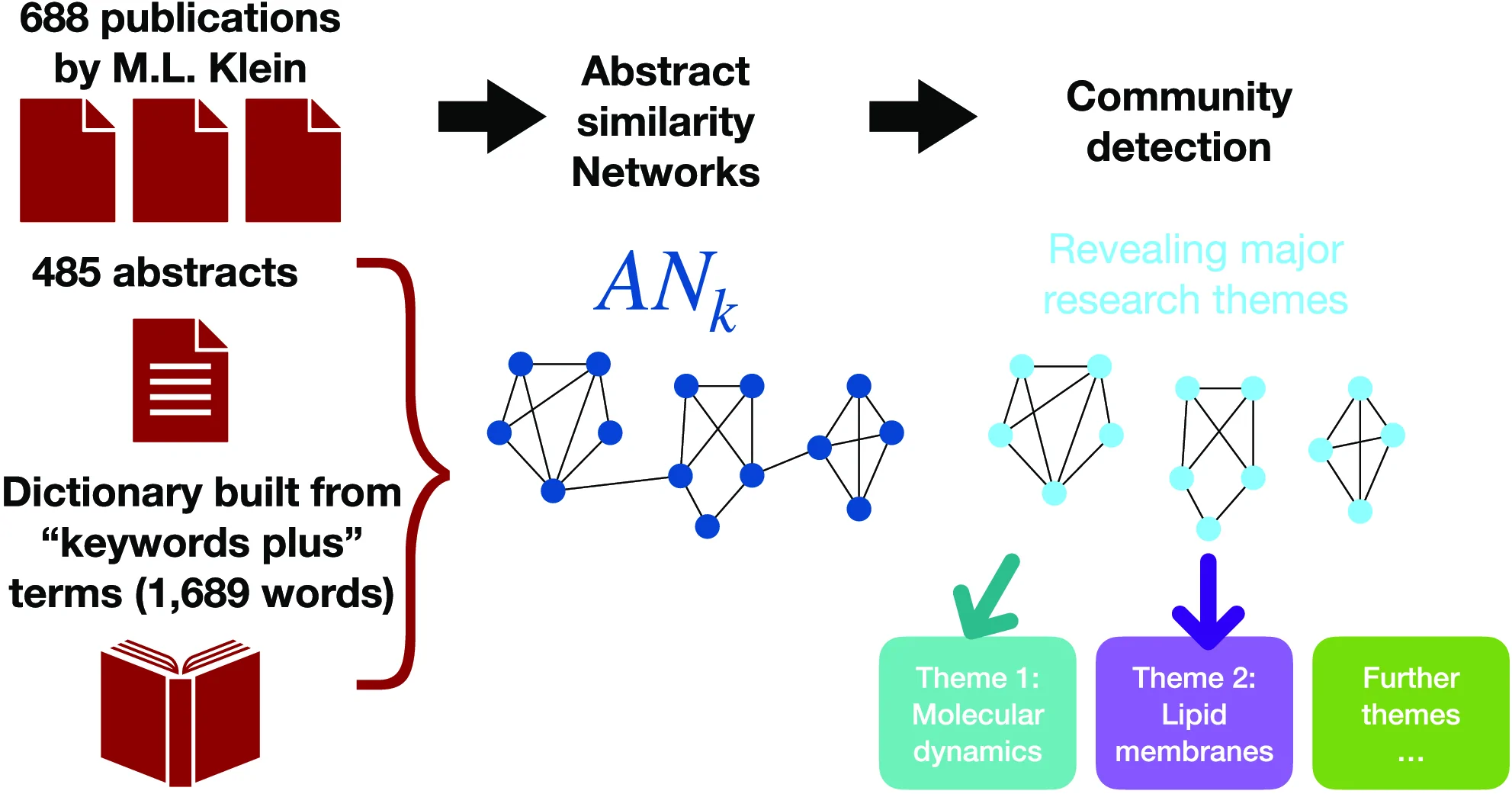

## 1 Introduction

Michael L. Klein is a leading figure in theoretical and computational chemistry whose research has helped define several major directions in molecular simulation.^1^ His scientific contributions extend from foundational developments in simulation methodology, such as thermostats and integrators,^2^ path-integral algorithms,^3^ free-energy and enhanced-sampling techniques,^4^ as well as to force-field development,^5^ particularly in developing transferable coarse-grained models for surfactants, lipids, and related soft materials. His research targeted a strikingly broad range of chemical and biological systems from aqueous systems^6^ and ionic solutions,^7^ to membranes^8^ and macromolecules,^9^ inspiring numerous research communities.

The combination of methodological innovation and thematic breadth makes Klein’s publication record especially well suited to bibliometric and network-based analysis. Similar studies have increasingly been used to identify influential contributions and reveal the internal structure of a scientific field, including recurring themes, methods and applications, and how collaborations and shared concepts organize and connect scientific fields. Dictionary-based approaches have long been employed to quantify semantic similarity between words, texts, and documents. Rather than relying exclusively on lexical overlap, these methods exploit external lexical resources to capture semantic relationships among textual units.^10,11^ Recently, Banerjee et al.^12^ studied the occurrence of the top 160 keywords provided by SCOPUS database across publications to analyze changes in trends within Cheminformatics/QSAR papers from 2000 onward compared to the previous years. Our scientiometric analysis takes advantage of employing graph theory and thus allows us to study not only the occurrence or frequency of specific terms, but their co-occurrence and topical relationship. For this, we construct a domain-specific dictionary directly from the entire keywords plus vocabulary extracted from the Web of Science records. Using this dictionary, we establish a family of abstract-similarity networks, enabling the investigation of relationships among publications from a graph-theoretical perspective rather than through pairwise similarity measures alone. Our previous scientometric studies have included coauthorship network analyses of research related to the Soai autocatalytic reaction^13^ and fluorous biphasic systems,^14^ together with keyword co-occurrence network analysis to reveal the thematic organization of molecular chirality research.^15^

In this study, we examine Klein’s scientific legacy through the lens of coauthorship networks and keyword co-occurrence patterns, with the aim of highlighting both the structure of his research community and the thematic connectivity of his work. For this purpose, we compiled a dataset of 688 research outputs indexed in the Web of Science as of 20 May 2026. We note that Michael L. Klein has a higher number of scientific publications because the number of publications indexed in the Web of Science database is generally lower than that in other databases, such as Google Scholar. This is due to the stricter indexing criteria applied by the Web of Science, which covers a more limited range of scientific document types. Furthermore, the Web of Science core collection provides comprehensive citation indexing only from 1975 onward, thus Klein’s works spanning the previous decade is missing from the current analysis. This means that approximately 70 publications, covering his earlier theoretical work on solids, such as on lattice dynamics of noble gases, self-consistent phonons, and the highly cited study of second-order elastic constants under stress^16^ is not included.

## 2 Basic Graph Theoretical Notions

A finite undirected network is simple, if it contains neither loops nor parallel edges; otherwise, it is called a multigraph. The simplified version of a network containing parallel edges is defined as the network obtained by replacing all parallel edges with single edges.

The two most important centrality measures examined in this study are defined as follows. Let *G* be a multigraph with vertex set *V* = {1, 2, 3, ···, *n*}, and edge set *E*. The degree (or degree centrality) of a vertex *i*, denoted by deg­(*i*), is the number of edges incident to it. (Loops are counted twice, but we have no loops in the networks of this work.) Degree primarily measures local embeddedness and immediate relational activity of the vertices, high degree suggesting strong local influence. The eigenvector centrality of an arbitrary vertex *i* is obtained in the following way.^17^ Let λ denote the largest positive eigenvalue of the adjacency matrix **A** of the graph *G*, that is a nonnegative symmetric square matrix with elements *A*_*ij*_ = 0, if there is no edge joining vertices *i* and *j* of the graph, and *A*_*ij*_ = *p*, if there are exactly *p* edges joining vertices *i* and *j*. Next, consider the corresponding eigenvector **x** with the property that its maximum coordinate is equal to 1. The eigenvector centrality *x*(*i*) of a vertex *i* is defined as the *i*-th coordinate of the eigenvector **x**. The correctness of the definition of the eigenvector centrality is based on an extension of the Perron-Frobenius theorem to square matrices with non-negative elements, that guarantees the existence of the eigenvector **x** with the above properties.^18,19^ Eigenvector centrality measures the structural importance of the vertex within the entire network, considering not only the direct connections but also their quality. A vertex has high eigenvector centrality precisely when it is connected to other vertices that themselves occupy important positions within the network. In a coauthorship multigraph, high-degree vertices represent authors incident to a large number of coauthorship edges, with repeated collaborations between the same pair of authors counted separately, whereas eigenvector centrality assigns higher scores to authors connected to other authors with high eigenvector centrality.

The modularity matrix of the network with *n* vertices and *m* edges, is a matrix **B** = (*B*_*ij*_)_*i*_^*n*^_*,j =* 1_ with elements $B_{\mathrm{ij}}=A_{\mathrm{ij}}-\frac{\deg\left(i\right)\cdot \deg\left(j\right)}{2m}$ . Communities of a network are densely connected subgraphs of the network. All the communities of the network in this paper were found using the leading eigenvector algorithm of the igraph package,^18^ based on the leading eigenvector method developed by M. E. J. Newman,^19–21^ as follows. The vertices are assumed to be organized into *K* communities, where 1 ≤ *K* ≤ *n*. The community indicator function is defined as δ­(*i*, *j*) = 1, if vertices *i* and *j* belong to the same community, and 0 otherwise. The modularity associated with a given community structure is defined in^19–21^ as $\frac{1}{2m}\sum_{i,j=1}^{n}B_{\mathrm{ij}}\delta \left(i,j\right)$ , where *n* and *m* denotes the number of vertices and edges, respectively. The leading eigenvector algorithm,^18–21^ based on the computation of the largest positive eigenvalue of the matrix **B** (with parallel edges included), iteratively partitions the network assigning vertices to communities according to the signs of the corresponding coordinates of the associated leading eigenvector. This subdivision process continues only when it produces a positive increase in modularity associated with the community structure. In brief, the leading eigenvector algorithm identifies community structure through the spectral decomposition of the modularity matrix, where the leading eigenvectors reveal the principal patterns of community organization within the network.

## 3 The Klein Network and Its Largest Community

The coauthorship network of publications authored by Michael L. Klein, hereafter referred to as the Klein network, is a multigraph containing parallel edges and comprising 801 vertices and 8771 edges. The vertices represent the authors of Michael L. Klein’s 688 scientific publications indexed in the Web of Science database^22^ (see Supporting Information). Two vertices in the Klein network are linked by *p* edges (*p* ≥ 1), if and only if the respective authors coauthored exactly *p* scientific publications by Michael L. Klein, listed in the Web of Science database.

The average degree of the Klein network is 21.9; a simplified version of the network is shown in Figure 1, where circles are the vertices, each representing one of the 801 authors. M. L. Klein is highlighted in red and the top 10 highest degree coauthors (see Table 1) are shown in blue (note that the spatial proximity of the vertices or their relative arrangement in the figure do not carry information). Gray lines are edges, showing coauthorship. For a network with such high average degree and vertex set size, representation of parallel edges using edge weighting would not be feasible in the figure, hence a single line is used irrespective of the number of shared publications. Nevertheless, all our calculations and interpretations take parallel edges into account, since parallel edges represent repeated collaborations or recurring occurrences. In the case of Michael L. Klein, these repeated connections (i.e., parallel edges) clearly indicate that a single joint paper most often gave rise to further scientific collaborations and long-term scientific relationships, continuing to foster new interactions in the future.

**Figure 1:**
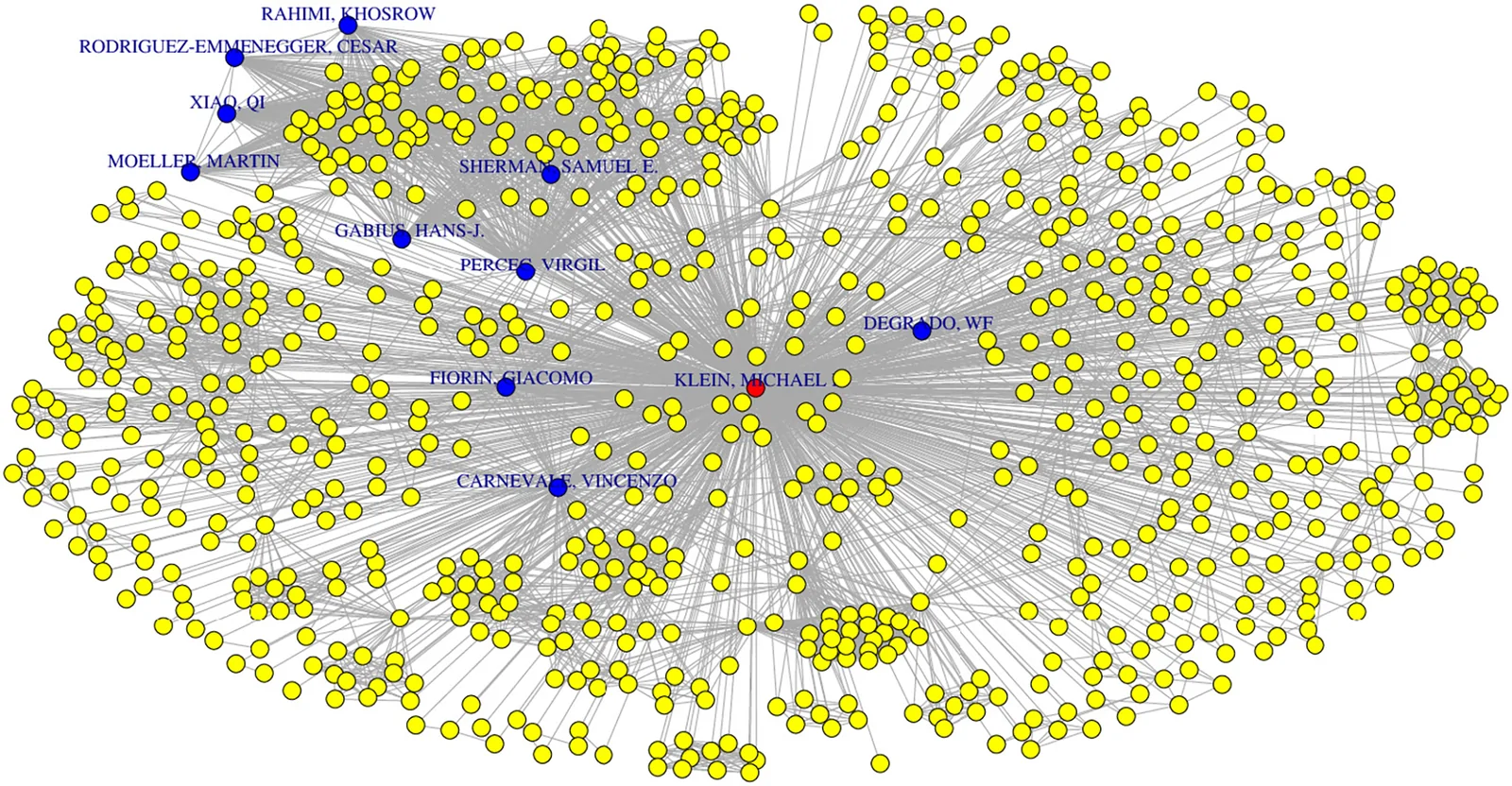
Simplified version of the entire Klein network. M. L. Klein is highlighted in red and the top 10 highest degree coauthors are shown in blue. Parallel edges are not displayed.

**Table 1:** Ten Co-Authors with the Highest Degree in the Klein Network and Their Eigenvector Centrality with the Corresponding Rank Shown in Parentheses

	Name	Degree	Eigenvector centrality
	M. L. Klein	2331	1.0000 (1)
1	V. Percec	344	0.3007 (2)
2	Q. Xiao	259	0.2365 (5)
3	V. Carnevale	233	0.3004 (3)
4	H.-J. Gabius	151	0.1387 (15)
5	W. F. DeGrado	145	0.1939 (10)
6	S. E. Sherman	139	0.1370 (16)
7	G. Fiorin	134	0.1974 (9)
8	C. Rodriguez-Emmenegger	127	0.1212 (20)
9	M. Moeller	122	0.1048 (28)
10	K. Rahimi	122	0.1120 (24)

All figures in this study were created using the igraph package for the R programming language (version 2.2.1, 2025). The vertices of the Klein network represent a large group of scientists that evolved over decades of sustained and productive collaboration around the scientific achievements of Michael L. Klein.

In this network, the ten coauthors of M. L. Klein with the highest degree are presented in Table 1. The last column of the table lists the eigenvector centralities, with the rank of each vertex in the network based on the eigenvector centrality shown in parentheses. Five of the scientists (on top of M. L. Klein) listed in Table 1 are not only in the top ten within vertices with the highest degree but also in the top ten within the highest eigenvector centrality, showing particular importance in the coauthorship network. These are V. Percec, Q. Xiao, V. Carnevale, W. F. DeGrado and G. Fiorin.

The largest numbers of parallel edges (number of shared publications) in the Klein network occur between the author pairs from Table 2. Only the ten author pairs connected by the largest numbers of parallel edges are included. Not surprisingly, M. L. Klein is a member of all ten pairs. He coauthored the largest numbers of publications with I. R. McDonald (46), V. Carnevale (42), and M. Sprik (35).

**Table 2:** Top Ten Author Pairs with the Largest Numbers of Parallel Edges in the Klein Co-Authorship Network

	Author pairs	Number of parallel edges
1	M. L. Klein–I. R. McDonald	46
2	M. L. Klein–V. Carnevale	42
3	M. L. Klein–M. Sprik	35
4	M. L. Klein–P. Moore	30
5	M. L. Klein–V. Percec	30
6	M. L. Klein–W. Shinoda	28
7	M. L. Klein–W. F. DeGrado	25
8	M. L. Klein–G. Fiorin	25
9	M. L. Klein–R. Devane	24
10	M. L. Klein–R. C. Remsing	23

Research shared with these coauthors cover a wide range of topics with strong overlap across themes–particularly those focusing on biomaterials–albeit some patterns emerges. The majority of publications with I. R. McDonald concentrate on solid phases and their wide range of structural properties, from rotator phases in alkanes to ionic solids, while research shared with M. Sprik focus on water, ionic solvation and interfaces, with ab initio and path integral methods providing a link between them. Publications with V. Percec form a collaboration cluster centered on dendrimers and synthetic membrane mimics, with a clear focus on supramolecular self-assembly and biomimetic interfaces. Publications with Q. Xiao appear more tightly focused on dendrimers, while works coauthored with V. Carnevale spans a methodologically more varied space from ion channels and ligand modulation, also representing a connection to the fundamental modeling of ionic solutions. The study of biomolecular structure and function, particularly metal-protein interactions are central to publications shared with W. F. DeGrado. Research coauthored with G. Fiorin covers topics from synthetic macromolecules to understanding the structure of ionic solvation shells, also including method and software development.

Beyond identifying Klein’s most frequent direct collaborators, it is also informative to examine the strongest coauthorship ties among his coauthors themselves, as these relationships help reveal the internal structure of the broader coauthorship network. Based on the Web of Science dataset,^22^ the three author pairs with the largest numbers of joint publications are V. Percec–Q. Xiao, R. Devane–W. Shinoda and W. F. DeGrado–G. Fiorin, with 21, 17, and 12 jointly authored publications, respectively. These most frequent coauthor pairs also map onto distinct topical clusters within the network, reflecting shared interest and complementary expertise, naturally. The V. Percec–Q. Xiao connection is dominated by work on dendrimersomes, glycodendrimersomes, membrane mimics, and glycan-mediated recognition; the R. Devane–W. Shinoda pair by coarse-grained modeling of surfactants, lipids, and self-assembly; and the W. F. DeGrado–G. Fiorin pair by membrane-protein biophysics, particularly the influenza A M2 proton channel.

It is interesting to identify other parts of the coauthorship network that are unique and highly connected in another way, such as a clique. A clique in a network is a subgraph in which every vertex is connected to every other vertex. The Klein network has a maximum clique that contains 29 authors: I. Andricioaei, M. L. Klein, D. J. Wales, S. Vanni, S. Urban, F. Tofoleanu, J. C. Smith, M. Tarek, J. Selent, M. Roy, N. Lindow, M. J. Lemieux, A. Kolocouris, S. Khalid, M. A. Kasimova, H.-C. Hege, P. Gkeka, R. Friedman, L. Delemotte, C. Del Val, J. T. Deckman, A.-N. Bondar, Z. Cournia, N.-V. Buchete, G. Brannigan, D. Baum, B. Antonny, T. W Allen and J. Henin. This clique represents a single publication, a review article, for which large authorship is not uncommon, especially if it provides a broad and exhaustive overview of the current state of a field–in this case that of membrane protein structure and function, including both experimental and theoretical perspective.^23^ The maximum clique of the Klein network together with its parallel edges is shown in Figure 2.

**Figure 2:**
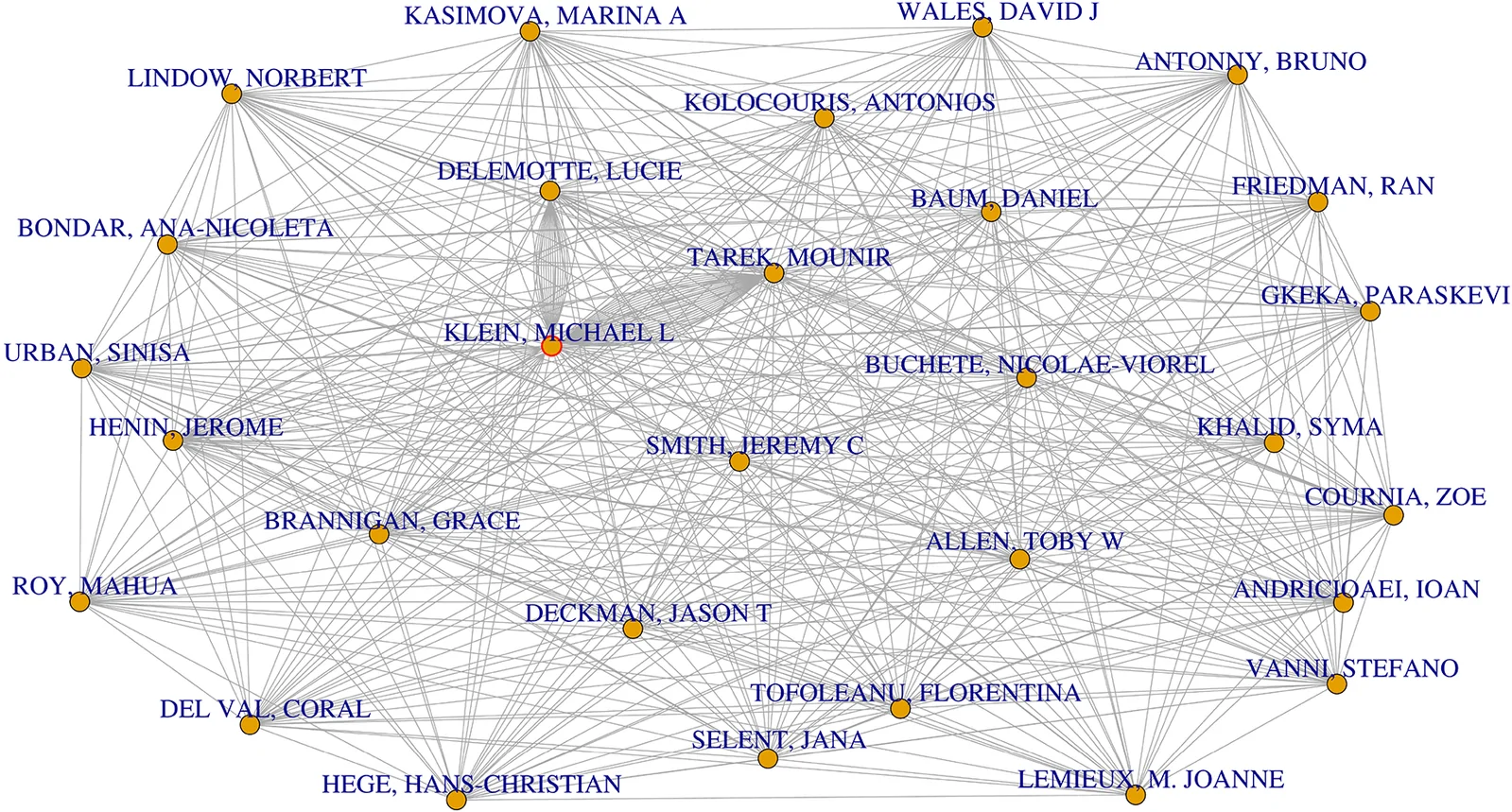
Maximum clique of the Klein network, showing the 29 authors. Parallel edges are displayed.

While this clique is determined by a single manuscript, several of its authors published further studies jointly together: the largest numbers of parallel edges in the maximum clique of the Klein network occur between the author pairs M. L. Klein–M. Tarek (22), M. L. Klein–L. Delemotte (15), M. L. Klein–J. Henin (9), M. L. Klein–G. Brannigan (7) and J. Henin–G. Brannigan (5). The latter confirms that authors J. Henin and G. Brannigan together have five joint publications with M. L. Klein in the Web of Science database.

Investigating the community structure of a coauthorship network can help identify the principal patterns of scientific collaboration. The detected communities may reveal groups of researchers who collaborate more intensively with one another than with the rest of the network, thereby highlighting the principal collaborative structures underlying the research domain. The leading eigenvector algorithm detected 16 different communities, consisting of 203, 120, 103, 66, 65, 59, 55, 40, 26, 15, 11, 10, 9, 7, 7, and 5 vertices (authors), respectively. The largest community contains 1266 edges. By replacing parallel edges with single edges for simpler visualization, its simplified version contains 538 edges and it is shown in Figure 3.

**Figure 3:**
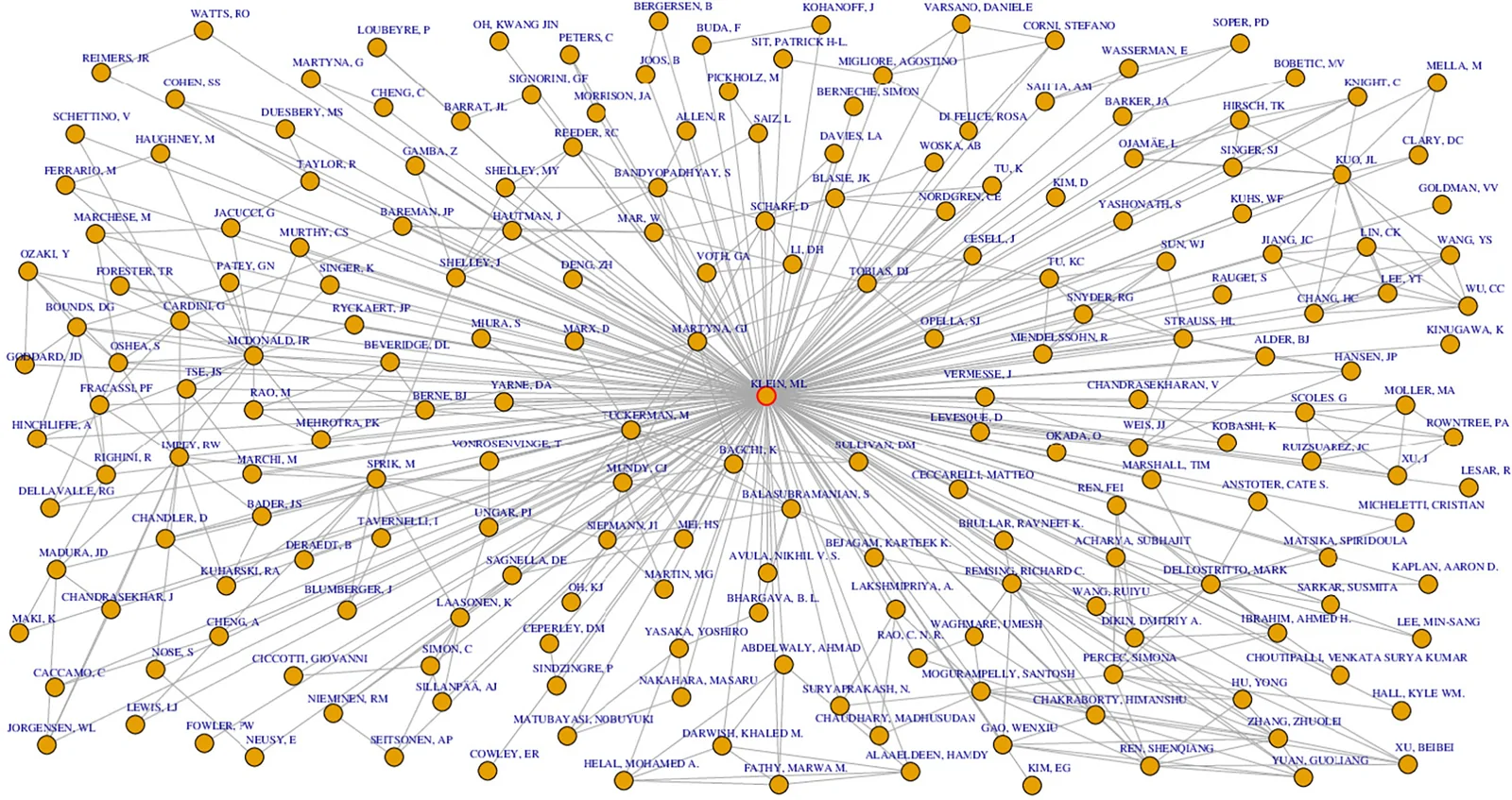
Simplified version of the largest community of the Klein network. For better visualization only 538 edges are shown, parallel edges are replaced by single ones, but all calculations take these repeated collaborations into account.

Among the authors listed in Tables 1 and 2, four belong to the largest community detected by the leading eigenvector algorithm (M. L. Klein, I. R. McDonald, M. Sprik, and R. C. Remsing), while six belong to the second-largest community (V. Percec, Q. Xiao, H.-J. Gabius, S. E. Sherman, C. Rodriguez-Emmenegger and M. Moeller). There are several publications in the database which share at least six coauthors from these lists: these papers typically focus on the role of cell-surface sugars (glycans) in molecular recognition and cellular communication. Collectively, they examine glycan-dependent binding, protein–glycan interactions, and the contribution of glycans to self-assembly,^24–26^ with the broader aim of clarifying structure–function relationships and guiding the design of artificial membrane-mimetic systems.^27,28^

## 4 Author Keywords of the Publications of Michael L. Klein

Papers that share a large number of author keywords generally address similar research topics and scientific questions. We examined the author keywords assigned to Michael L. Klein’s publications indexed in the Web of Science database and analyzed the relationships among these keywords. The author keywords were standardized to ensure consistency. Synonymous expressions were consolidated into a single keyword (e.g., coarse graining and coarse grain), and singular and plural variants were unified (e.g., cell membrane mimic and cell membrane mimics were considered identical keywords). Only 113 papers contained author keywords, from these we identified a total of 380 distinct expressions. Taken together, these keywords provide insights into recurring themes across the publications and may also indicate broader research directions and trends represented within the dataset.

The keyword network assigned to Michael L. Klein’s publications, presented in Figure 4, is constructed from the 380 author keywords, with each vertex representing a keyword and edges indicating keyword co-occurrence: two vertices (keywords) are linked by *p* ≥ 1 edges in this network if and only if they co-occur as keywords in exactly *p* different papers. This network is a multigraph containing 1072 edges; for more visibility, the higher the degree of a node (keyword), the larger its size and lettering, and only the keywords of at least degree 5 are listed by name in the network.

**Figure 4:**
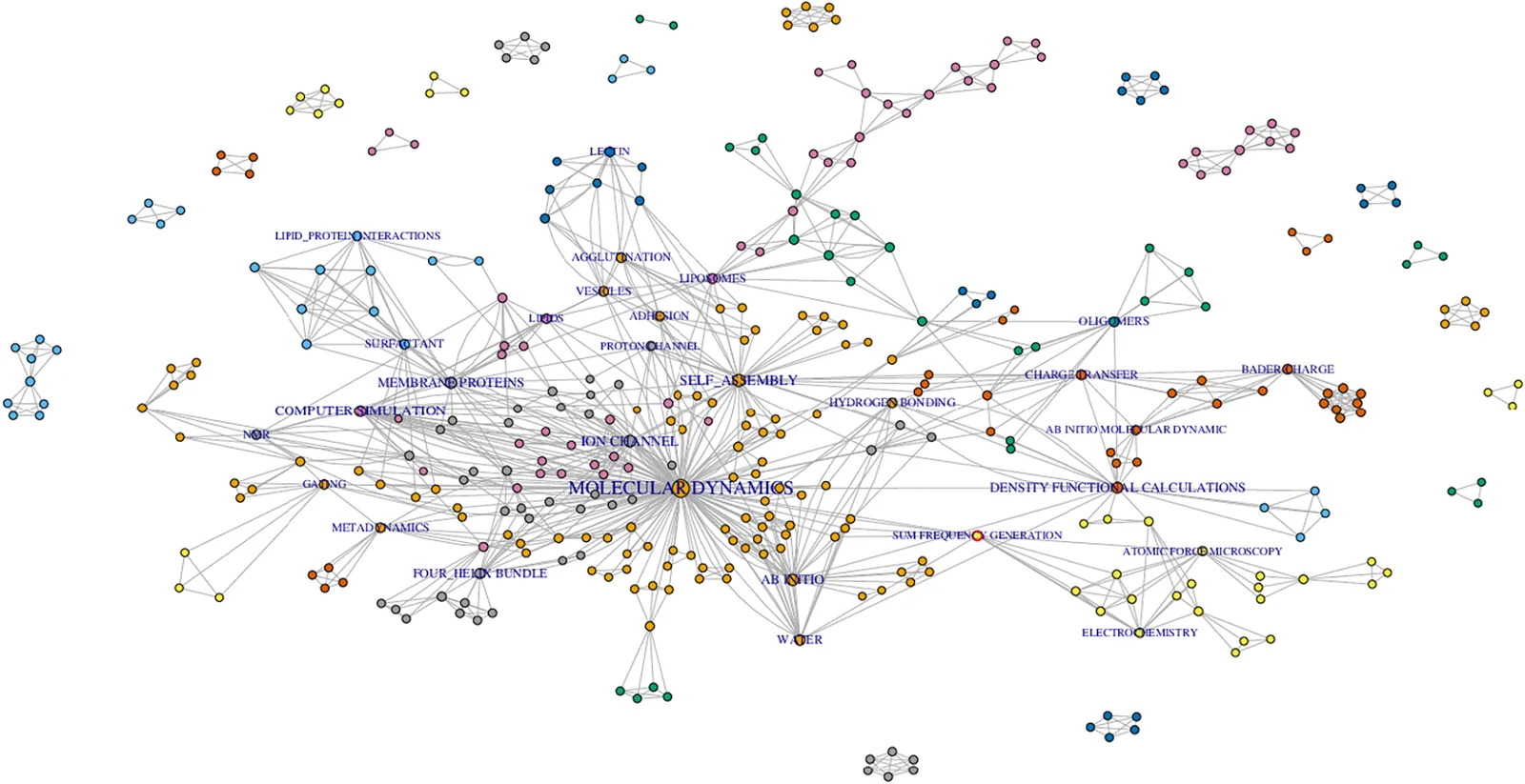
Keyword network assigned to Michael L. Klein’s publications: communities are shown in different colors and only keywords with a degree of 5 or higher are displayed by name.

Similar to the Klein coauthor network, the ten keywords with the highest degree in the keyword network are presented in Table 3. The last column of the table lists the eigenvector centralities, with the rank of each keyword in the network based on the eigenvector centrality shown in parentheses. Eight keywords appear in both the top 10 lists for vertices with the highest degree and those with the highest eigenvector centrality, namely molecular dynamics, self-assembly, ion channel, computer simulation, ab initio, membrane proteins, water and four-helix bundle–representing both the key topics and materials, as well as the range of fundamental computational methods within the database.

**Table 3:** Ten Keywords with the Highest Degree

	Keyword	Degree	Eigenvector centrality
1	molecular dynamics	152	1.0000 (1)
2	self-assembly	36	0.3282 (5)
3	ion channel	32	0.4594 (2)
4	computer simulation	28	0.3542 (4)
5	ab initio	27	0.3550 (3)
6	membrane proteins	26	0.1905 (8)
7	density functional calculations	23	0.1135 (21)
8	water	19	0.2788 (6)
9	four-helix bundle	17	0.2086 (7)
10	liposomes	14	0.1067 (23)

In the keyword network assigned to Michael L. Klein’s publications, the largest numbers of parallel edges occur between the keyword pairs from Table 4. The high frequency of the these keyword pairs is not unexpected, as computational studies often combine a keyword describing the primary method (e.g., molecular dynamics or ab initio), and one denoting the simulated system or material (e.g., water or ion channel), or refer to the main process or property under investigation (e.g., self-assembly or hydrogen bonding). In this sense, the most common parallel edges do not merely reflect repeated vocabulary, but reveal the characteristic way in which publications link simulation methodology to specific chemical or biological questions. By contrast, the adhesion-agglutination pair is less generic and points to a more specialized thematic cluster of work on membrane mimics, and cell-surface interactions, showing that this topic is particularly prominent in the studied publication portfolio.

**Table 4:** Keyword Pairs Linked with the Largest Number of Parallel Edges

	Keyword pairs	Number of parallel edges
1	ion channel–molecular dynamics	6
2	ab initio–molecular dynamics	5
3	computer simulation–molecular dynamics	4
4	molecular dynamics–self-assembly	4
5	molecular dynamics–water	4
6	four-helix bundle–molecular dynamics	3
7	ab initio–acidity	2
8	ab initio–hydrogen bonding	2
9	acidity–molecular dynamics	2
10	adhesion–agglutination	2

The leading eigenvector algorithm detected 38 different communities, the largest three of them consisting of 62, 37, and 28 vertices (keywords), respectively. The maximum clique contains the following 10 keywords: Bader charge, charge distribution, thermolysin, Mn-catalase, RNase H, metalloproteins, metal ions, Due Ferri four-helix bundle, charge transfer and metal site, all listed for a single publication, hence naturally forming a clique.

## 5 Comparison of Abstracts Based on a Specific Dictionary: The Abstract-Similarity Network *AN*_*k*_

While relatively few of the publications downloaded from the Web of Science records include author keywords (only 113), the vast majority of the articles (485 from the 688 papers) do have abstracts. The relationship between articles thus can also be examined based on their abstracts, and we performed a dictionary-based text similarity analysis between them, specifically. For this purpose, we first construct a dictionary from the 1,689 “keywords plus” terms contained in the 507 papers from the downloaded Web of Science data^22^ presented as a table in the Supporting Information. We emphasize that these keywords are not provided by the authors themselves, but are generated automatically by the Web of Science, based on terms that frequently occur in the titles of cited publications. In the present study, these bibliometrically relevant–often compound–keywords are used because they ensure keyword assignment for almost all publications (507 from the 688 papers) allowing us to examine connections among a much wider body of publications than in case of considering author keywords. Compound terms (e.g., cell membrane mimic) are retained in their original form and are treated as single keywords. Here, too, just as in the analysis of author keywords, singular and plural variants are unified. For simplicity, each paper was assigned an identification number corresponding to its row number in the table downloaded from the Web of Science database,^22^ and throughout this section we refer to publications by these row numbers.

For a fixed positive integer *k*, the abstract-similarity network *AN*_*k*_ is defined as a simple graph, as follows. Of the 688 publications, only those with available abstracts can be included as vertices in the network *AN*_*k*_. Consequently, the vertex set of the network *AN*_*k*_ can contain identifiers between 2 and 689, because the first row of the table is a header without any data. Vertices *i* and *j* are connected by an edge in the network *AN*_*k*_, if the corresponding abstracts share at least *k* words from the dictionary. Isolated vertices are removed; therefore, the vertex set of *AN*_*k*_ contains only those publications with abstracts that share at least *k* dictionary words with the abstract of at least one other publication.

Smaller values of *k* produce denser networks with more vertices and edges. For *k* = 1, the network *AN*_1_ contains 485 vertices (abstracts) and 87,748 edges. The leading eigenvector algorithm found two communities; the size of the larger one was 252. Network *AN*_2_ contains the same vertices, but only 51,149 edges. The leading eigenvector algorithm found three communities; the size of the largest one was 136. As *k* increases, fewer pairs of abstracts satisfy the condition of sharing at least *k* dictionary words, and the number of edges decreases accordingly. Because isolated vertices are omitted, the number of vertices also decreases as *k* increases, and the network breaks down into an increasing number of communities until *k* = 8. At the same time, larger values of *k* may yield to communities whose member papers exhibit greater abstract-level similarity.

The abstract-similarity networks *AN*_*k*_ were examined for *k* ≥ 3, Table 5 contains the main results. For each network, we recorded the main structural characteristics, including the number of vertices, the number of edges, the number of communities detected by the leading eigenvector algorithm, the size of the largest community, and the size of the largest clique.

**Table 5:** Structural Characteristics of the Abstract-Similarity Networks

*k* value	Number of edges	Number of vertices	Number of communities	Size of the largest community	Size of the maximum clique
3	25,826	477	4	141	53
4	11,782	457	5	156	24 (8 cliques)
5	5140	430	6	166	15
6	2122	363	8	122	12
7	923	283	15	82	8
8	388	200	28	54	6 (6 cliques)
9	163	120	23	27	5 (2 cliques)
10	72	76	21	13	3 (8 cliques)
11	40	58	21	12	3 (2 cliques)
12	21	31	12	6	3 (2 cliques)
13	8	11	4	5	3
14	4	7	3	1	0

Within each abstract-similarity network *AN*_*k*_, the largest community may also be interpreted from a spectral perspective, as it represents the most prominent community-level structure identified by the leading eigenvector algorithm. Figures 5, 6, and 7 depict the communities of networks *AN*_10_, *AN*_11_ and *AN*_12_. The largest community of the *AN*_10_ network, shown in light yellow in Figure 5, contains 13 vertices.

**Figure 5:**
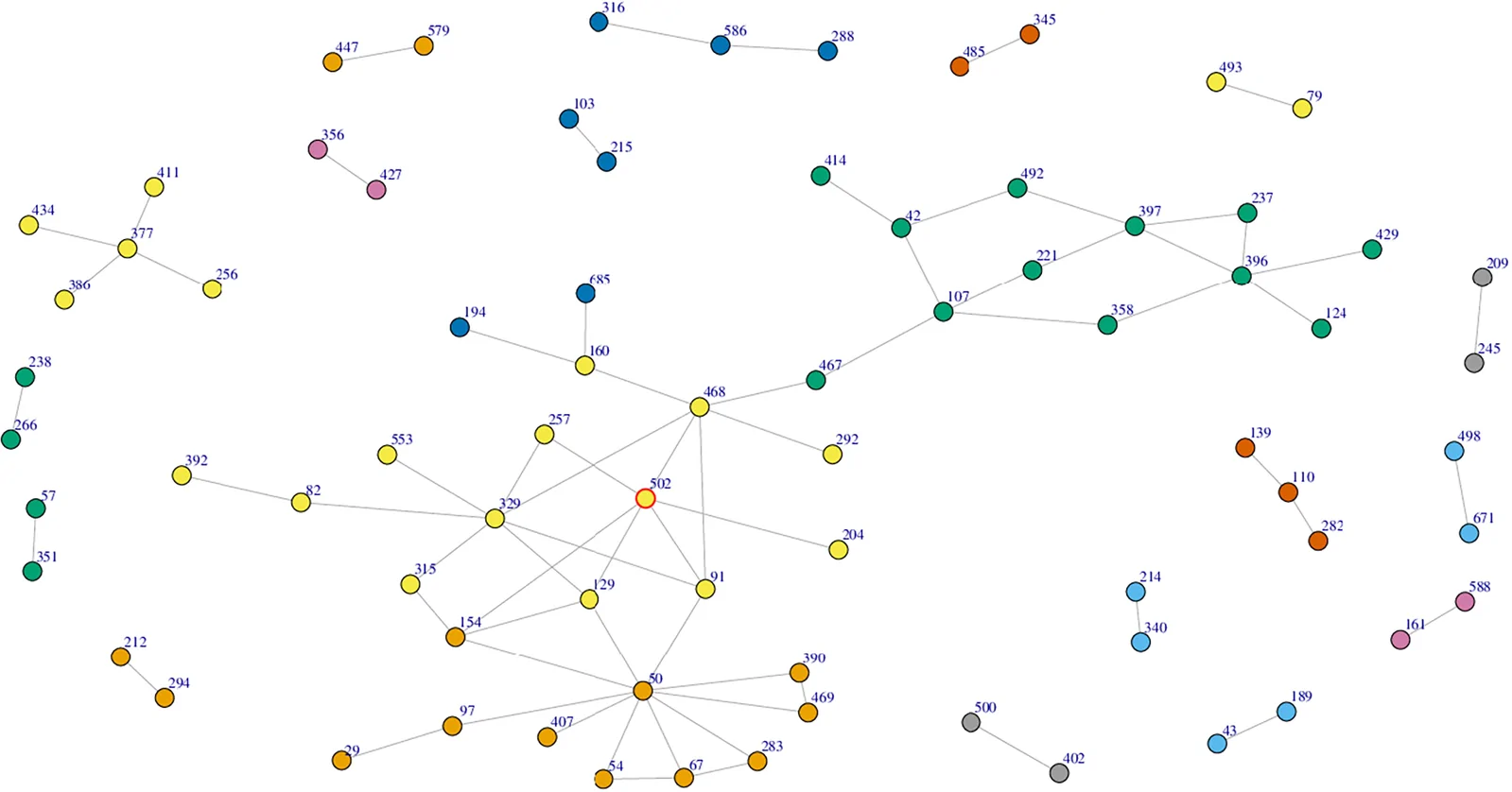
Communities of abstract-similarity network for *k* = 10. Papers are identified by their row numbers in the Web of Science download, and communities are shown in different colors.

**Figure 6:**
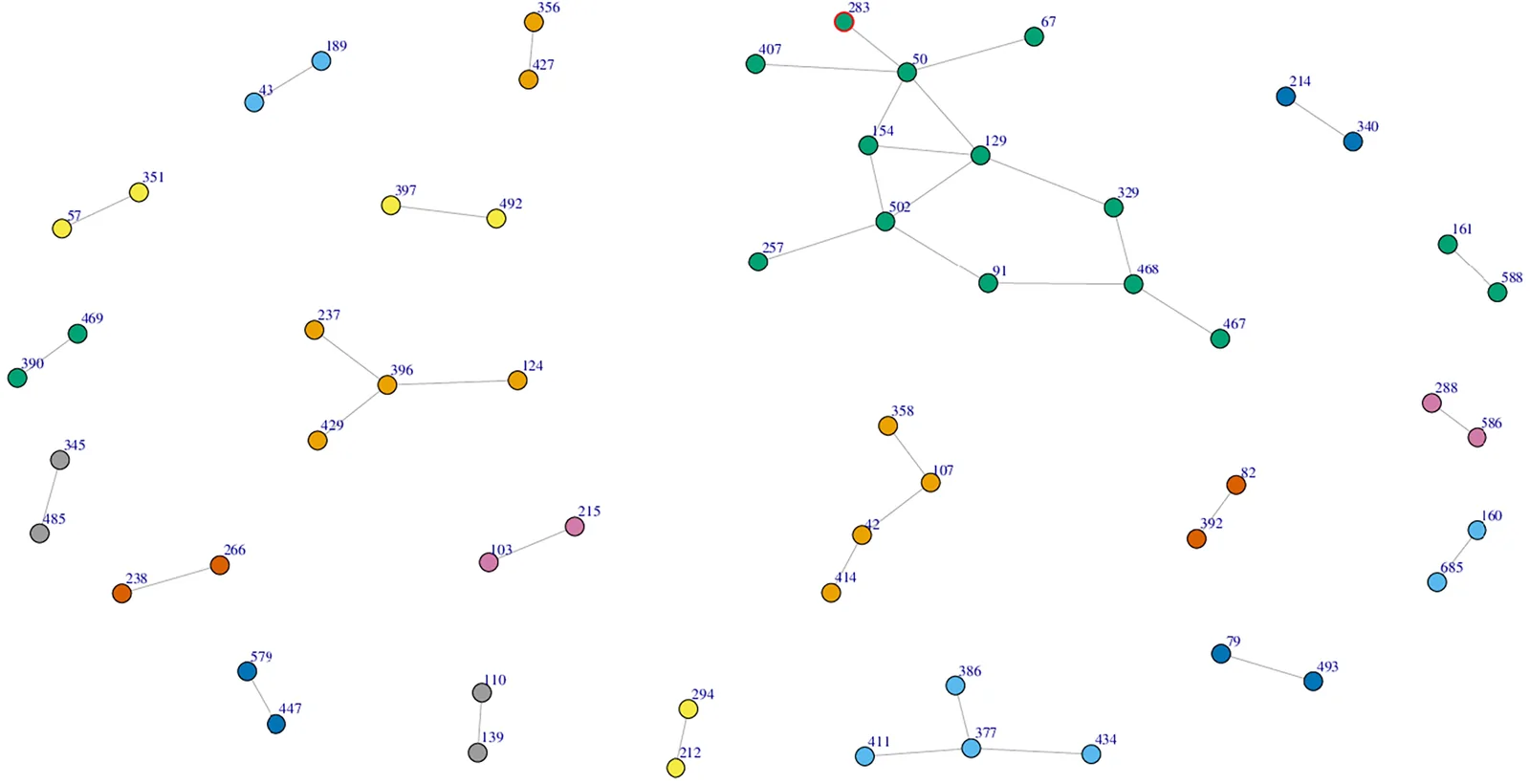
Communities of abstract-similarity network for *k* = 11. The detected communities coincide with the connected components for the first time. The largest community, highlighted in green, will provide more information later on.

**Figure 7:**
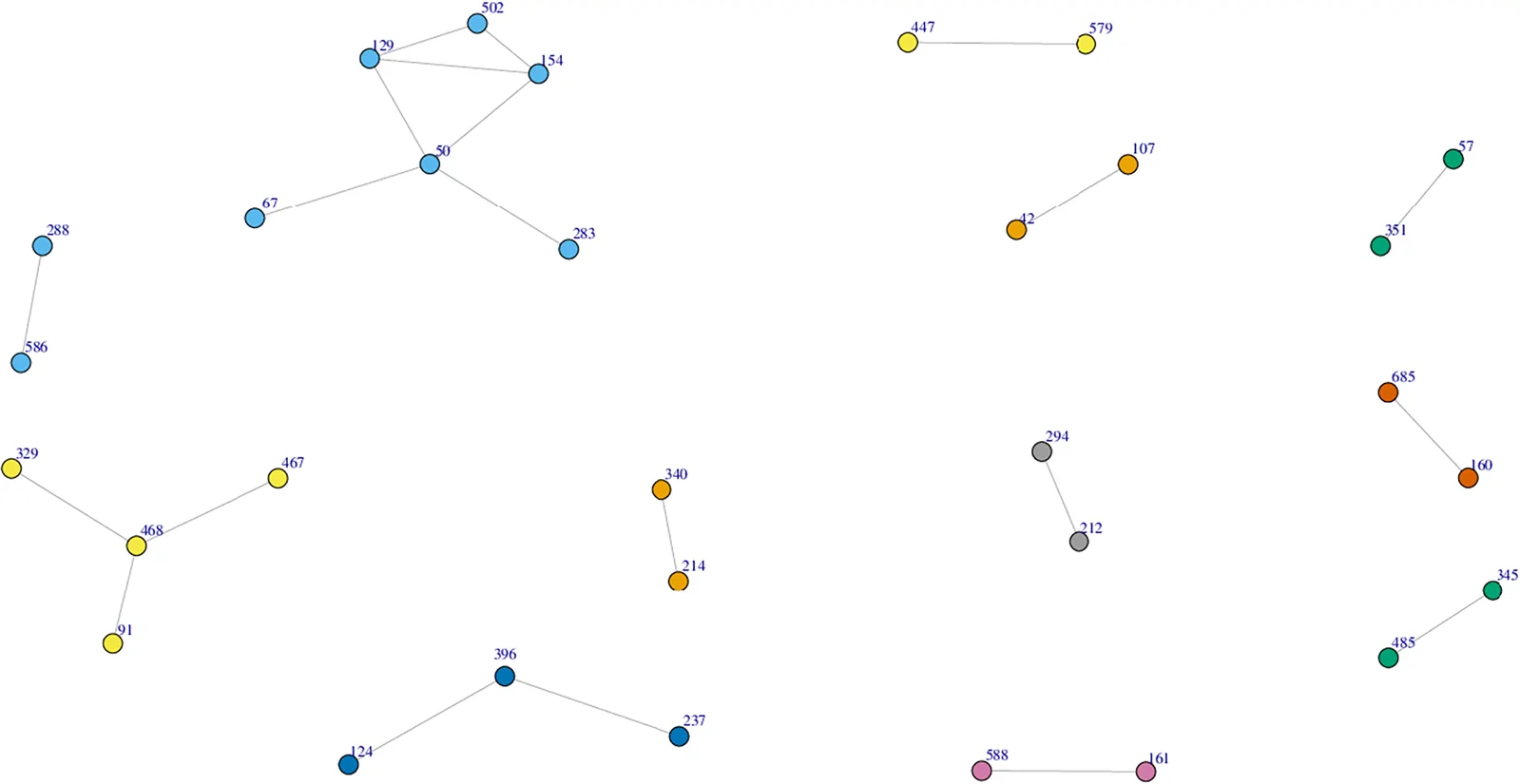
Communities of abstract-similarity network for *k* = 12.

For larger values of *k*, the network becomes progressively smaller and more fragmented, reflecting the fact that increasingly few pairs of abstracts share sufficiently many dictionary words. In network *AN*_11_, the detected communities coincide with the connected components for the first time. That is, the network has become sufficiently fragmented such that each connected component corresponds to exactly one community, with no component containing vertices from multiple communities. The largest community of the *AN*_11_ network, shown in green in Figure 6, contains 12 vertices, corresponding to the articles {50, 67, 91, 129, 154, 257, 283, 329, 407, 467, 468, 502}. Table 6 presents the authors and titles of these publications. Rather than representing a single highly specialized research topic, this community appears to encompass several closely related research directions, covering themes recurring across the wider publication database: molecular simulations of lipid membranes, integrating studies of membrane structure and anesthetic–membrane interactions. While our analysis extends beyond simply calculating the frequency of specific expressions, in this case it is also interesting to look at the keywords plus terms associated with these 12 articles directly. These articles have 70 distinct keywords plus terms, of which 20 are with multiplicities, as follows: keywords referring to membranes appear 19 times (lipid bilayer(8), membrane(4), phospholipid bilayer(5), phosphatidylcholine model membrane(2)), keywords referring to anesthetics occur 8 times (general-anesthetics(3), Meyer-Overton hypothesis(3), halothane(2)), with several further keywords referring to experimental measurements used for comparisons (magnetic-resonance(5), H-2 NMR(2), neutron diffraction(2)) and simulation methods (simulation(2), molecular dynamics simulation (2)).

**Table 6:** Articles Belonging to the Largest Communities of the Networks *AN*_*k*_ for *k* = 11, 12, 13, 14, Together with Their Identification Numbers

Identification number	Authors	Title of the article
50	K. Tu; D. J. Tobias; M. L. Klein	Constant pressure and temperature molecular dynamics simulation of a fully hydrated liquid crystal phase dipalmitoylphosphatidylcholine bilayer
67	K. Tu; D. J. Tobias; J. K. Blasie; M. L. Klein	Molecular dynamics investigation of the structure of a fully hydrated gel-phase dipalmitoylphosphatidylcholine bilayer
91	L. Saiz; M. L. Klein	Structural properties of a highly polyunsaturated lipid bilayer from molecular dynamics simulations
129	L. Koubi; M. Tarek; M. L. Klein; D. Scharf	Distribution of halothane in a dipalmitoylphosphatidylcholine bilayer from molecular dynamics calculations
154	K. Tu; M. Tarek; M. L. Klein; D. Scharf	Effects of anesthetics on the structure of a phospholipid bilayer: Molecular dynamics investigation of halothane in the hydrated liquid crystal phase of dipalmitoylphosphatidylcholine
257	M. Pickholz; L. Saiz; M. L. Klein	Concentration effects of volatile anesthetics on the properties of model membranes: A coarse-grain approach
283	T. Husslein; D. M. Newns; P. C. Pattnaik; Q. F. Zhong; P. B. Moore; M. L. Klein	Constant pressure and temperature molecular-dynamics simulation of the hydrated diphytanolphosphatidylcholine lipid bilayer
329	L. Koubi; L. Saiz; M. Tarek; D. Scharf; M. L. Klein	Influence of anesthetic and nonimmobilizer molecules on the physical properties of a polyunsaturated lipid bilayer
407	K. Tu; D. J. Tobias; M. L. Klein	Constant pressure and temperature molecular dynamics simulations of crystals of the lecithin fragments: Glycerylphosphorylcholine and dilauroylglycerol
467	L. Saiz; M. L. Klein	The transmembrane domain of the acetylcholine receptor: Insights from simulations on synthetic peptide models
468	L. Saiz; S. Bandyopadhyay; M. L. Klein	Effect of the pore region of a transmembrane ion channel on the physical properties of a simple membrane
502	L. Koubi; M. Tarek; S. Bandyopadhyay; M. L. Klein; D. Scharf	Effects of the nonimmobilizer hexafluoroethane on the model membrane dimyristoylphosphatidylcholine

Publications 91, 129, 257, 329, 468, and 502 are included in the largest communities of both abstract-similarity networks *AN*_10_ and *AN*_11_. These six publications are connected not only by the shared methodology (molecular dynamics) but considering the structure of an interface and the effect of electrostatic interactions in influencing local molecular distributions and orientations. Interestingly, in five of the papers the studied interface is of a lipid bilayer, while in one case (publication 91) is the surface of alkali chloride solutions.

The largest community of the *AN*_12_ network, shown in blue in Figure 7, is a subset of cardinality 6 of the largest community of network *AN*_11_ namely the set {50, 67, 129, 154, 283, 502}. These papers again share the methodology (molecular dynamics) and the studied system (lipid bilayer, in five cases it is also specified to be in the liquid-crystal phase), however, the abstracts are further connected by the specification that the simulations were performed at constant pressure (which is standard in atomistic simulations of membranes) and that the simulation results are compared to experimental measurements. We mention here that connections between the vertices of abstract-similarity networks strongly depend on the level of details the authors provide in the abstracts.

In the last two abstract-similarity networks, *AN*_13_ and *AN*_14_, the leading eigenvector algorithm detected only four and three communities, respectively. The largest community in *AN*_13_ has vertex set {50, 67, 129, 154, 283}, whereas the largest community in *AN*_14_ has vertex set {50, 67, 283}. Both sets are subsets of the largest community of network *AN*_11_. Figure 8 depicts the *AN*_13_ and *AN*_14_ networks, and Table 6 lists (among others) the authors and titles of the publications belonging to their largest communities. The three publications of the {50, 67, 283} set are closely connected, studying the hydrated liquid crystal phase and structure of the dipalmitoylphosphatidylcholine lipid bilayer specifically, focusing on the structure and ordering of the lipid tails.

**Figure 8:**
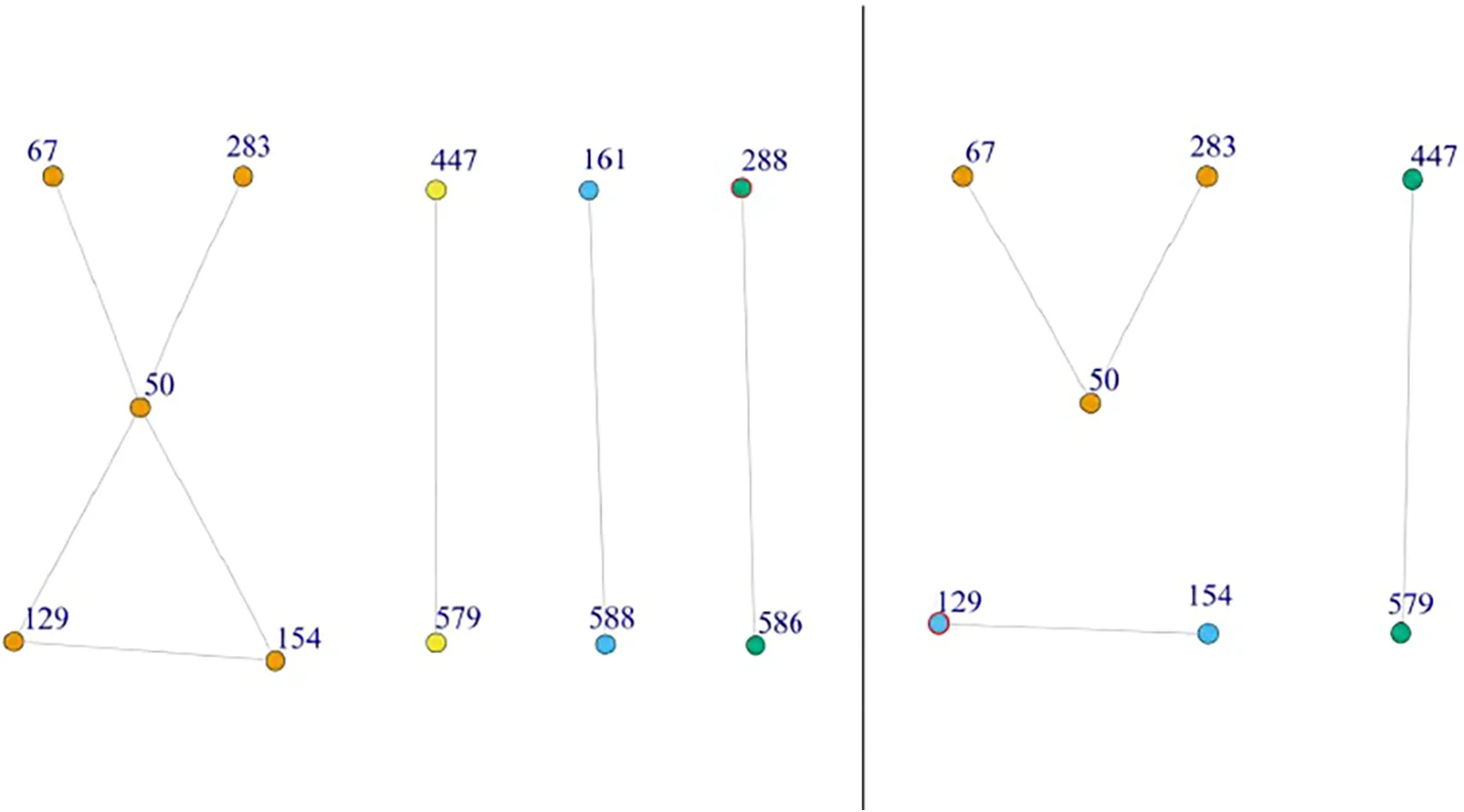
Communities of abstract-similarity networks for *k* = 13 (left side) and *k* = 14 (right side).

Just as the community structure changes, the clique structure also changes substantially as *k* increases. Papers corresponding to vertices of maximum cliques of *AN*_10_, *AN*_11_, *AN*_12_, *AN*_13_ and *AN*_14_, which are not included in Table 6, are contained in Table 7. For *k* = 10, the *AN*_10_ network contains eight maximum cliques, all of size 3, namely {237, 396, 397}, {50, 390, 469}, {50, 67, 283}, {50, 54, 67}, {91, 329, 468}, {50, 129, 154}, {129, 154, 502}, and {91, 468, 502}. For *k* = 11, only two maximum cliques of *AN*_10_ remain, namely {50, 129, 154} and {129, 154, 502}. The authors and titles of the publications corresponding to the above numbers are listed in Table 6 and Table 7. Several of these cliques include the same publications as identified earlier in communities, showing strong overlap between these two levels of organization. Some cliques also reveal more specific common themes; for example, publications {237, 396, 397} all focus on potassium ion channels in membranes.

**Table 7:** Articles Belonging to the Maximum Cliques of the Networks *AN*_*k*_ for *k* = 10, 11, 12, 13, 14, Which Are Not Listed in Table 6, Together with Their Identification Numbers

Identification number	Authors	Title of the article
54	K. Tu; M. L. Klein; D. J. Tobias	Constant-pressure molecular dynamics investigation of cholesterol effects in a dipalmitoylphosphatidylcholine bilayer
237	V. Carnevale; W. Treptow; M. L. Klein	Sodium Ion Binding Sites and Hydration in the Lumen of a Bacterial Ion Channel from Molecular Dynamics Simulations
390	L. Saiz; M. L. Klein	Influence of highly polyunsaturated lipid acyl chains of biomembranes on the NMR order parameters
396	S. Vemparala; C. Domene; M. L. Klein	Computational Studies on the Interactions of Inhalational Anesthetics with Proteins
397	C. Domene; S. Vemparala; S. Furini; K. Sharp; M. L. Klein	The role of conformation in ion permeation in a K+ channel
469	R. G. Snyder; K. Tu; M. L. Klein; R. Mendelssohn; H. L. Strauss; W. J. Sun	Acyl chain conformation and packing in dipalmitoylphosphatidylcholine bilayers from MD simulation and IR spectroscopy

The *AN*_12_ has the same two maximum cliques. Thus, paper triples {50, 129, 154} and {129, 154, 502} represent particularly stable and strong abstract-based relationships within the Web of Science dataset under increasingly restrictive similarity criteria. Publications in these two cliques are all included in the largest community we identified in *AN*_11_ as well.

## 6 Conclusions

Through this study, we celebrate the enduring and still evolving scientific contribution of Michael L. Klein. Our analysis of the coauthorship network reinforces the fundamentally collaborative nature of scientific research and shows that Klein has not only contributed seminal ideas, but has also helped shape and sustain a remarkably broad scientific community that continues to develop around his work. The network structure indicates that recurrent collaborators often support the growth of specific research directions, leading to dense clusters of shared publications, while at the same time helping connect otherwise distinct areas, particularly through methodological innovation and force-field or model development.

The author-keyword network suggests that computational methods and simulation methodology act as important thematic bridges across otherwise diverse application areas. However, as the majority of publications do not have author keywords, it was necessary to find other means to establish thematic connections. In the present work we introduce abstract-similarity networks (485 of 688 publications had abstracts), using the complete list of keywords plus expressions as our dictionary of scientifically relevant words. The resulting abstract-similarity networks point to broad conceptual connections across Klein’s publications, highlighting the extent to which methodological development, molecular modeling, and applications to complex materials and biomolecular systems are interwoven throughout his research career, with the largest identified communities centered around lipid membranes.

Our results also show that long-term or repeated coauthorship is not a prerequisite for high impact: for example, Klein’s publication with W. L. Jorgensen, J. Chandrasekhar, J. D. Madura, and R. W. Impey^29^ does not appear as a dominant feature in any of our networks, yet it remains one of the most influential papers in atomistic modeling of water and is the most highly cited publication in his body of work (37,462 citations in the Web of Science database, as well as being the second most cited paper of the journal, as of July 2026). Since neither author keywords nor keywords plus terms are assigned to this article, furthermore, the Web of Science database does not include its abstract, this paper does not appear in the keyword graph or in the abstract similarity networks, highlighting possible shortcomings of relying solely on data available in the Web of Science. Publications with very short abstracts (i.e., including fewer words) or those including fewer scientifically relevant details are less likely to appear in large communities of the abstract similarity networks *AN*_*k*_, due to lower probability of sharing multiple keywords plus terms with other abstracts. This can result in highly influential works (such as refs 2 and 30) to not appear as prominent in our analysis. Identified topical relationships can also be sensitive to the level of detail or context provided in the abstract. For example, most membrane simulations are carried out on the isobaric ensemble, yet only a subset of abstracts state this explicitly, which can artificially strengthen links among those papers; similarly, some abstracts refer to broader long-term motivations or application contexts, such as relevance to membrane systems, even when these are not the direct focus of the study.

In summary, our approach combines two methodological elements. First, a corpus-specific dictionary is generated automatically from keywords plus terms of the investigated publication set, eliminating the need for external lexical resources. Second, publication abstracts are analyzed through this dictionary and graph theoretical perspective rather than providing statistics of keyword occurrence. Although the proposed methodology was demonstrated using Michael L. Klein’s publications, its applicability is broader and provides a reference for similar future studies, also highlighting the importance of using a robust and complete database. Future studies including temporal analysis of the networks could provide further insights into how emerging methods enable new applications or how the scientific focus evolve within a research field.

## Supplementary Material



## Notes

Table of publications authored by Michael L. Klein (Web of Science Core Collection Search, downloaded on 20 May 2026) (XLS)
